# Enduring Power of Print: How Health Information Pamphlets Promote Equity and Trust in Patient Education

**DOI:** 10.1200/OP-25-00739

**Published:** 2025-10-17

**Authors:** Diana C. Dima, Aman Sium, Meredith Giuliani, Janet Papadakos

**Affiliations:** ^1^ Cancer Education, Princess Margaret Cancer Centre, University Health Network, Toronto, ON, Canada; ^2^ The Institute for Education Research, University Health Network, Toronto, ON, Canada; ^3^ Radiation Medicine Program, Princess Margaret Cancer Centre, University Health Network, Toronto, ON, Canada; ^4^ Department of Radiation Oncology, University of Toronto, Toronto, ON, Canada; ^5^ Institute for Health Policy, Management & Evaluation, University of Toronto, Toronto, ON, Canada

Patients take an increasingly active role in cancer care, and patient education plays a crucial role in improving self-management, adherence to treatment, and health outcomes.^1^ In recent years, digital technologies have transformed health education and care delivery, bringing both potential and pitfalls in health equity and education.^2,3^ Health care institutions are increasingly offering online and asynchronous patient education and support, ranging from websites to e-books and eLearning modules and including video and audio materials. However, recent years have also seen the increasing online proliferation of health misinformation and disinformation, including during health crises like the COVID-19 pandemic.^4,5^ How can patient education best reach a diverse population while remaining accessible and future-proof, and what is the role of print materials in a digital world? Here, we evaluate recent evidence about patients' learning preferences and follow-up in previous work^6^ by discussing the opportunities and challenges associated with the use of pamphlets in patient education today.

We suggest that a multimodal approach to patient education, combining print materials and online resources, can encourage equity and ensure that patients have access to resources they can easily find, navigate, and trust. Online education provides important advantages and will certainly keep growing as more aspects of cancer care shift from hospitals to homes; but we argue that pamphlets remain important in an overwhelming information landscape, even for people with good digital skills.

## IMPROVING EQUITY IN PATIENT EDUCATION

The migration of health care systems and health education to online platforms has often been framed as a step toward accessibility and equity. Asynchronous, freely available education can indeed reach people with limited time and resources or who live in underserved areas; yet it is becoming more and more clear that such advances can also leave people behind. Technological innovations often target those with the knowledge and resources to access them and are expected to eventually trickle down to the general population, yet end up widening inequities.^7,8^ Because of the pervasiveness of digital health care, digital literacy has become a critical social determinant of health.^9,10^

Globally, access to the Internet remains unequally distributed. In 2024, 32% of the global population did not use the Internet, particularly in developing countries. This population was also more likely to be older, lower-income, and less educated.^11^ Thus, despite ongoing efforts to promote digital access and literacy, accessing online resources remains a challenge for a significant proportion of population. Lack of digital literacy tends to intersect with other axes of marginalization, such as socioeconomic status and education,^12-14^ making it all the more important to ensure the inclusivity of patient education interventions.

In health care, systemically marginalized communities are often disadvantaged by technological advances, as shown by disparities in the use of and access to digital patient systems by racialized populations.^15,16^ Digital redlining is pervasive and may be more difficult to address with new technologies whose impacts are often underevaluated.^17^

Print material, on the other hand, remains accessible to patients who may struggle to seek and evaluate health information online. Pamphlets are readily available in health care institutions, libraries, and schools; they do not require digital skills or knowing what information to seek and where to seek it; and they do not impose time or cost restrictions on patients. Thus, a multimodal approach that supplements online resources with print material remains most effective in providing equitable patient education.^18^

## FIGHTING OVERWHELM AND MISINFORMATION

**FIG 1.**
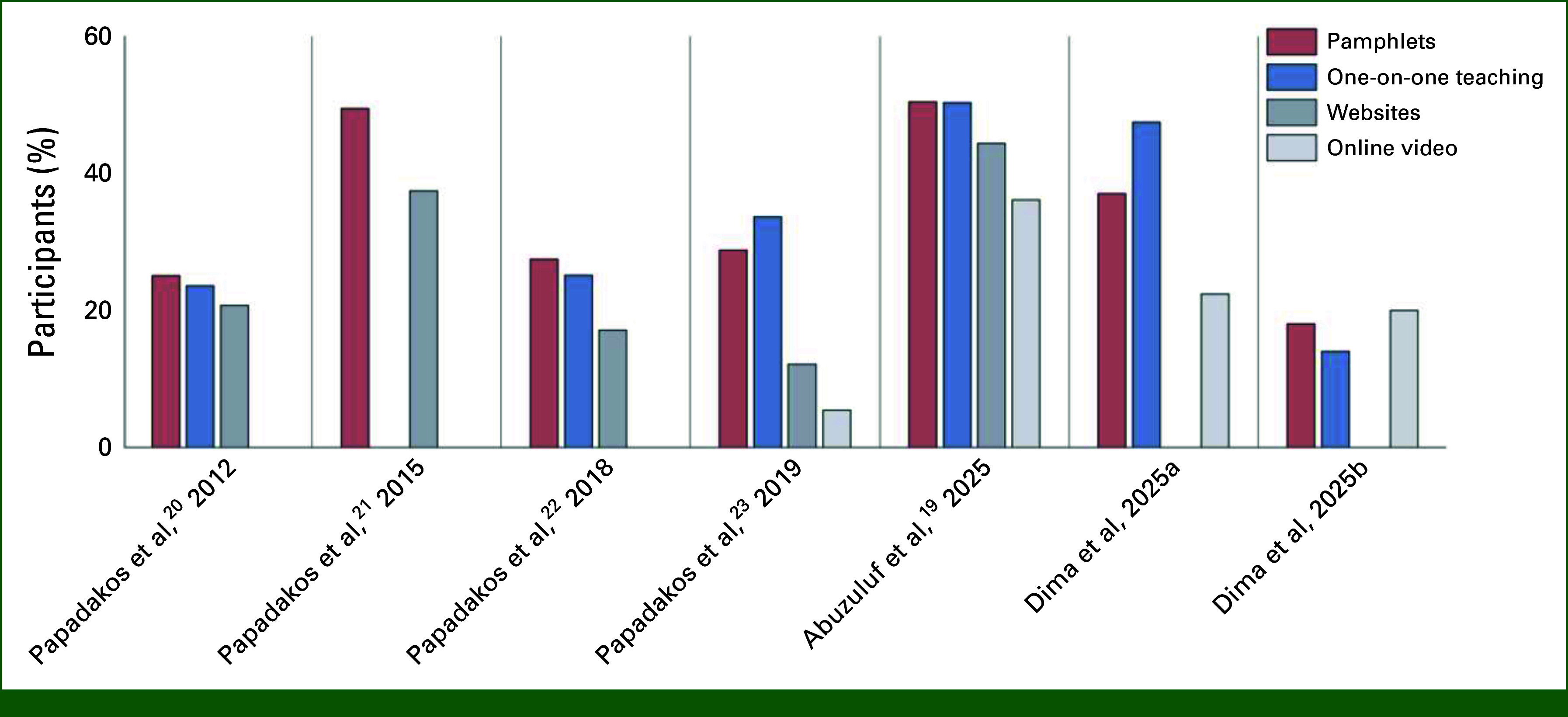
Information modality preferences among patients with cancer and caregivers across the years: results from a series of studies conducted at the Princess Margaret Cancer Centre in Toronto, Ontario. Despite differences in design and analysis, different samples between 2012 and 2025 rate pamphlets as their preferred or second most preferred method of health information delivery: Papadakos et al,^20^ 2012: gynecologic cancer survivors (N = 85); Papadakos et al,^21^ 2015: patients with gastrointestinal cancer (N = 82); Papadakos et al,^22^ 2018: patients with head and neck cancer (N = 445); Papadakos et al,^23^ 2019: patients with brain metastases (N = 106); Abuzuluf et al,^19^ 2025: patients with lung cancer and caregivers (N = 103); Dima et al 2025a: adolescent and young adult cancer survivors and caregivers (N = 76, in press); Dima et al 2025b: cancer caregivers (N = 115, in press).

Pamphlets are highly popular even with patient populations that benefit from Internet access and strong digital skills. A series of informational needs assessments in the largest cancer center in Canada between 2012 and 2025^19-23^ has shown time and time again that pamphlets remain a preferred way of receiving information. Pamphlets almost always rank above online materials, including websites and videos, and are only sometimes second to one-on-one teaching by health care professionals (Fig 1). Notably, the majority of patients in these studies report having reliable Internet access and score highly on validated computer proficiency scales.

This suggests that print material will not be rendered obsolete as digital access and literacy increase. In contrast, pamphlets may appeal because of all the ways in which they are different from online material: the fact that they are compact and tangible remains unchanged, they can be easily referred to at any time, and they are provided by trustworthy sources such as health care institutions. Research shows that health care professionals remain the primary and most trusted source of information for patients.^24-26^ Given the time constraints health care professionals face, pamphlets are a useful complementary source of information that patients can trust.

Patients often report being overwhelmed with information and needing help to navigate resources or even avoiding information related to their diagnosis.^27-29^ Online media are particularly likely to cause information overload because of the many sources of information competing for patients' attention.^30^ This effect is compounded by patients' limited health literacy, which remains an issue worldwide: even in developed countries with high levels of education, some 50% of adults have limited health literacy.^31^ This can make it difficult to seek, navigate, and evaluate health information and is often associated with low digital literacy.^33,34^ Pamphlets presenting health information in plain language^35^ can help overcome this barrier. By presenting patients with digestible information relevant to their needs, pamphlets can reduce overwhelm and minimize the effort needed to find and filter information.

Alongside its ever-increasing quantity, the quality of medical information available online has long been recognized as a serious challenge.^36^ Health misinformation is rampant online, particularly on social media,^4,5,37^ and disinformation campaigns have already resulted in decreasing vaccination rates and preventable outbreaks. In recent years, distrust in institutions and health care professionals has been rapidly rising.^4,37^ Technologies based on large language models bring about their own pitfalls, in some cases allowing misconceptions to spread and widening disparities.^38-40^ In recent informational needs assessments, patients with cancer and caregivers overwhelmingly ask for information provided directly by health care professionals, either one-on-one at appointments or via pamphlets.^19^ Pamphlets containing reliable, compact, and static information can thus be an antidote to the overwhelming and fast-paced online information landscape.

Although reliable and accurate online resources are increasingly being provided by health care institutions, pamphlets have the advantage of also being highly accessible. They are often provided at appointments by health care professionals and are thus part of the patient's journey without requiring additional steps. This ensures that essential information reaches those who are unlikely to seek it or find it elsewhere. Digital pamphlets are also often available online via health institutions' websites, ensuring that patients have access to trusted information in their preferred format.

## ENHANCING COMPREHENSION AND MEMORY RETENTION

While some of the advantages of health information pamphlets, such as compactness and reliability, translate to their digital counterparts, there are particular benefits to tangible print resources that patients can take home. First, the ability to revisit material at their own pace can help patients absorb new and complex health messages. While patients might forget, struggle to find, or get distracted while visiting online resources, tangible pamphlets may be more likely to be remembered and shared with family members.

Second, research highlights a strong advantage for print material in education. People learn and remember information better when presented in print, particularly when reading non-narrative texts.^41^ Despite the prevalence of online learning materials, this effect has been replicated over decades, and even young populations such as university students state a preference for printed materials.^42^ To effectively communicate key health messages at a critical time for patients and ensure optimal information retention, print remains unparalleled.

## CHALLENGES FOR PRINT RESOURCES

Given the low levels of public health literacy, it is essential that patient education materials, whether print or online, are readable and effective. Recent work suggests that the readability of both online and print health information remains limited, with most resources exceeding the recommended reading level.^43-45^ It is thus a priority to ensure that health information is communicated in plain language, following the best principles of accessible design and readability.^8,35^ Particular consideration must be given to the curation and design of information in such compact formats, which may not easily allow for a nuanced presentation of information and cannot be updated as easily as online resources. Furthermore, language and communication style should be consistent across materials, enabling patients to easily connect information.^46^

Finally, making print materials available in different languages can be challenging. Digital health information is more likely to be translated because of reduced cost, and machine translation, while still imperfect, improves accessibility.^47-50^ Digital materials may also be more accessible to people using screen readers and other accessibility tools. It is thus essential to ensure that health information pamphlets are available both digitally and in print. Digital advances and print resources can work together in providing effective health information to as many people as possible.

## TOWARDS EQUITABLE PATIENT EDUCATION

Evidence from informational needs assessments and qualitative surveys shows that print materials remain important in patient education as trusted, compact, and accessible sources of essential information. To best reach and educate patients without widening existing disparities in health outcomes, it is essential to provide learning materials in multiple formats.

Online materials can be effective, engaging, and accessible, providing asynchronous access to a wealth of resources; yet for groups that lack digital access, skills, or support, print materials are preferable. Print materials also enhance information retention and can help move learning away from the online medium, which can be difficult to navigate and rife with misinformation. Thus, a choice of modalities can foster effective learning at a time when learning is particularly difficult as patients may be overwhelmed and struggling during their cancer journey.

In line with best practice recommendations, multimodal patient education remains essential in delivering key health messaging in health care institutions. Recent results suggest that this is unlikely to change despite the increase in digital resources and access. As competing sources of information and misinformation struggle for the public's attention online, even digitally literate patients report a preference for print material. In a rapidly shifting information landscape, pamphlets create endless opportunities for trustworthy, personalized communication of health information that can reach people across demographic and socioeconomic barriers.
